# Novel epiphytic root-fungus symbiosis in the Indo-Pacific seagrass *Thalassodendron ciliatum* from the Red Sea

**DOI:** 10.1007/s00572-024-01161-9

**Published:** 2024-07-29

**Authors:** Martin Vohník, Jiřina Josefiová

**Affiliations:** 1 Department of Mycorrhizal Symbioses, Institute of Botany, Czech Academy of Sciences, Průhonice, Czechia; 2 KROKODIVE.CZ, Údolní 219/47, Prague, 14700 Czechia; 3 Laboratory of Molecular Biology and Bioinformatics, Institute of Botany, Czech Academy of Sciences, Lesní 322, Průhonice, 25243 Czechia

**Keywords:** Seagrasses, Marine fungi, Root-fungus symbioses, Dark septate endophytes, Epiphytism, Nutrient uptake, Necromass decomposition, Blue carbon sequestration

## Abstract

Symbioses with fungi are important and ubiquitous on dry land but underexplored in the sea. As yet only one seagrass has been shown to form a specific root-fungus symbiosis that resembles those occurring in terrestrial plants, namely the dominant long-lived Mediterranean species *Posidonia oceanica* (Alismatales: Posidoniaceae) forming a dark septate (DS) endophytic association with the ascomycete *Posidoniomyces atricolor* (Pleosporales: Aigialaceae). Using stereomicroscopy, light and scanning electron microscopy, and DNA cloning, here we describe a novel root-fungus symbiosis in the Indo-Pacific seagrass *Thalassodendron ciliatum* (Alismatales: Cymodoceaceae) from a site in the Gulf of Aqaba in the Red Sea. Similarly to *P. oceanica*, the mycobiont of *T. ciliatum* occurs more frequently in thinner roots that engage in nutrient uptake from the seabed and forms extensive hyphal mantles composed of DS hyphae on the root surface. Contrary to *P. oceanica*, the mycobiont occurs on the roots with root hairs and does not colonize its host intraradically. While the cloning revealed a relatively rich spectrum of fungi, they were mostly parasites or saprobes of uncertain origin and the identity of the mycobiont thus remains unknown. Symbioses of seagrasses with fungi are probably more frequent than previously thought, but their functioning and significance are unknown. Melanin present in DS hyphae slows down their decomposition and so is true for the colonized roots. DS fungi may in this way conserve organic detritus in the seagrasses’ rhizosphere, thus contributing to blue carbon sequestration in seagrass meadows.

## Introduction

Seagrasses, “the whales of the plant world”, are the only vascular plants that have returned to a fully submerged life in the marine environment. Their ancestors were among basal lineages of the brackish Alismatales that appeared more than 100 Mya, during the Cretaceous (Larkum et al. 2018). With ca. 70 described species, seagrasses represent a minor part of the total vascular plants’ diversity, yet they play indispensable roles in nutrient cycling, maintaining coastal ecosystems’ biodiversity and integrity, and blue carbon (= organic carbon sequestered in coastal and marine ecosystems) storage. They occur in coastal areas of all continents except Antarctica and their underwater meadows are among the most productive ecosystems on Earth, storing as much organic carbon per unit area as terrestrial forests (Fourqurean et al. 2012). In forests, most plants depend on the nutrient uptake through fungal hyphae (i.e., through mycorrhizal symbioses), plant necromass decomposition is to a large extent governed by fungi (Osono 2007; Boddy and Watkinson 1995; Read et al. 2004), and plants invest significant amounts of the photosynthetically fixed carbon to the underground mycelium of their mycorrhizal mycobionts (Hawkins et al. 2023). However, despite many reports on the diversity of marine fungi associating with seagrasses (e.g., Sakayaroj et al. 2010; Mata and Cebrián 2013; Ettinger and Eisen 2019; Vohník 2022), next to nothing is known about their functioning and how they contribute to the ecosystem services provided by seagrasses, including blue carbon storage.

Fungi are a diverse clade of eukaryotic organisms inhabiting almost all terrestrial, freshwater, and marine ecosystems. They represent a substantial part of the microbial diversity on Earth and play a key role in global biomass turnover and major food webs, comprising important predators, parasites, pathogens, and beneficial symbionts of many organisms. Fungi have interacted with plants long before terrestrialization and the resulting symbioses are among the oldest and most important associations on our planet (Naranjo-Ortiz and Gabaldón 2019). Arguably the most significant and widespread terrestrial plant-fungus symbioses are mycorrhizae, lichens, and various foliar and root endophytic associations. Symbioses with fungal endophytes (= fungi colonizing plant tissues without causing damage) evolved as a part of the defense system against herbivores, pathogens, and drought stress, thus enhancing plant growth (Saikkonen et al. 1998; Arnold 2007) and occur in most vascular plants. Mycorrhizae evolved as means of nutrient uptake, occur in most vascular land plants, and their predecessors probably facilitated plant terrestrialization (Selosse and Le Tacon 1998; Brundrett 2002). The most ancient and by far the most common mycorrhizal type is arbuscular mycorrhiza (AM), “the mother of plant root endosymbioses” (Parniske 2008) occurring in ca. 74% of vascular plant species, incl. many freshwater, salt marsh, and mangrove plants (van der Heijden et al. 2015). On the other hand, while extant Alismatales comprise a mixture of non-mycorrhizal and AM families, the four seagrass families (Cymodoceaceae, Hydrocharitaceae, Posidoniaceae, and Zosteraceae) are regarded as non-mycorrhizal (Brundrett 2017).

The absence of mycorrhizae in seagrasses is not surprising, as they can take up nutrients from the water column through the leaves and from the seabed through the non-mycorrhizal roots (Short and McRoy 1984; Terrados and Williams 1997). However, many marine ecosystems are oligotrophic, resulting in the leaf uptake being unable to cover all nutritional needs, and several seagrasses form organic seabed sediments storing large amounts of nutrients that are not directly accessible to non-mycorrhizal roots. The prime example is *Posidonia oceanica* (Posidoniaceae) dominating the mostly oligotrophic Mediterranean Sea (Powley et al. 2017) that forms “matte”, an organo-mineral seabed sediment which can be several meters thick and hundreds to thousands of years old (Serrano et al. 2012), sometimes being referred to as soil (Piñeiro-Juncal et al. 2020). Indeed, matte in a way resembles peat and peatland ecosystems gave rise to a specific type of mycorrhizal symbiosis that is formed by fungal symbionts (= mycobionts) phylogenetically close to saprobic fungi (Fehrer et al. 2019; Rice and Currah 2006). Intriguingly, also *P. oceanica* hosts a specific root-fungus symbiosis (Vohník et al. 2015) that is morphologically similar to the association formed by the dark septate endophytes (DSE) in the roots of most terrestrial plants (Jumpponen and Trappe 1998). DSE are a miscellaneous group of mostly sterile ascomycetous mycobionts that in some cases facilitate host’s nutrient uptake (Usuki and Narisawa 2007; Newsham 1999), hence some authors include them among mycorrhizal fungi (Jumpponen 2001). The symbiosis is formed by *Posidoniomyces atricolor* that represents an independent marine biotrophic lineage in the Aigialaceae family (Pleosporales), its closest relatives being plant-associated saprobes from marine, terrestrial, and freshwater habitats in Southeast Asia and Central America (Vohník et al. 2019).

Except *P. oceanica*, no other seagrass is known to form any similar root-fungus symbiosis. On the other hand, only a small fraction of seagrasses has been thoroughly examined for colonization by fungi, so one may wonder how many novel symbioses, perhaps formed by hitherto undescribed mycobiont lineages, await discovery. The factors favoring seagrass symbioses with fungi that improve nutrient uptake include seagrass species that produce high amounts of biomass (i.e., with high nutrient demands) and oligotrophic conditions with most mineral nutrients bound in recalcitrant (typically organic) substrates. In other words, they would be more likely to evolve in highly productive seagrasses occurring in oligotrophic waters with a seabed containing organic detritus (e.g., seagrass necromass). Thus, it may come as no surprise that we recently discovered an association formed by a dark septate (DS) mycobiont in the roots of *Thalassodendron ciliatum* (Cymodoceaceae), a highly productive seagrass in the mostly oligotrophic Red Sea that produces dense root mats accumulating organic matter (Lipkin 1979). In this paper we describe its anatomy and morphology using stereomicroscopy, and light and scanning electron microscopy. In addition, we used DNA cloning followed by Sanger sequencing to search for the mycobiont forming this novel marine symbiosis.

## Materials and methods

### Sampling

**Fig. 1:**
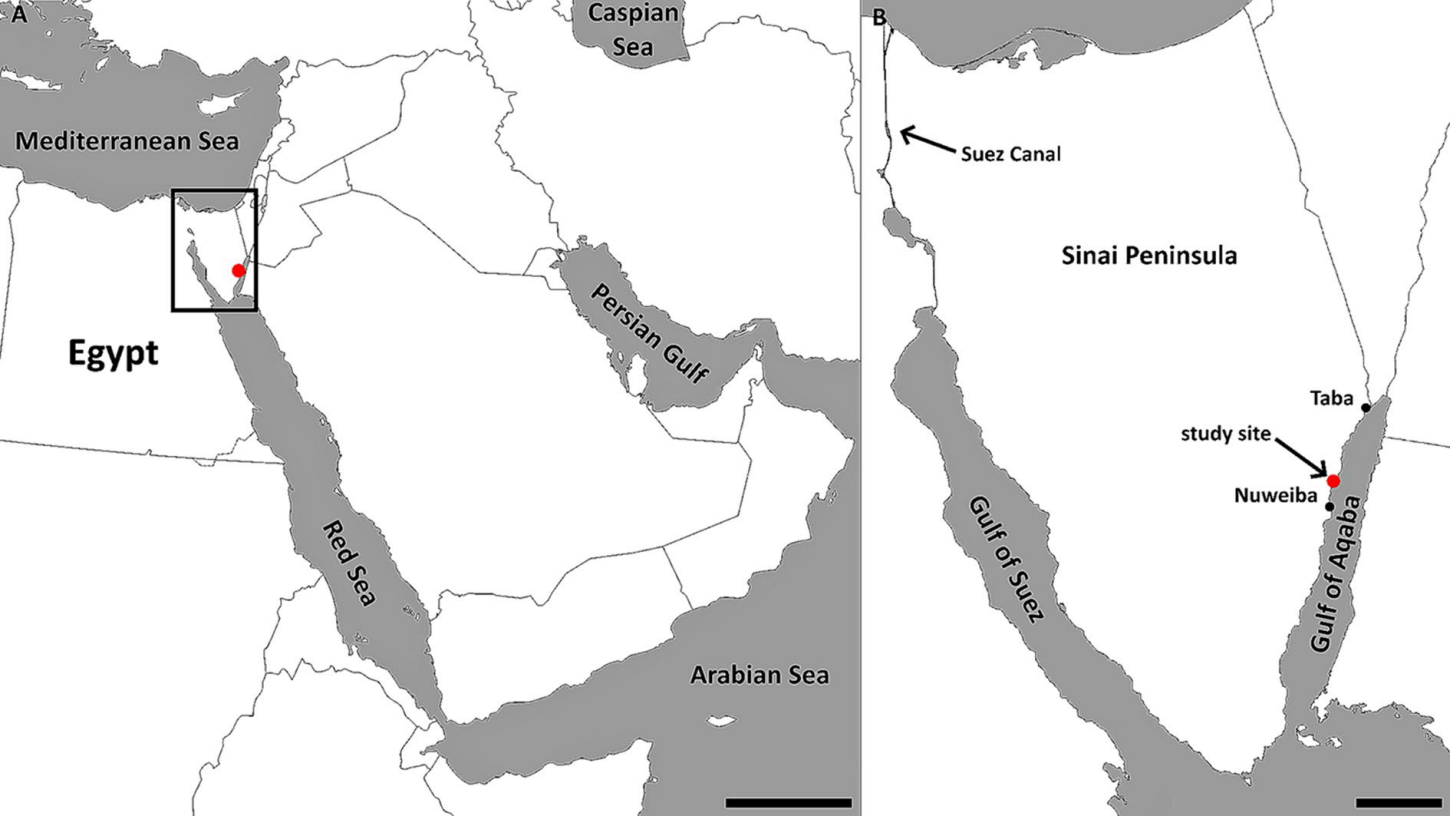
Location of the study site in the Middle East. The rectangle in (**A**) delimits the area depicted in (**B**). Bars represent 500 km and 50 km, respectively

**Fig. 2:**
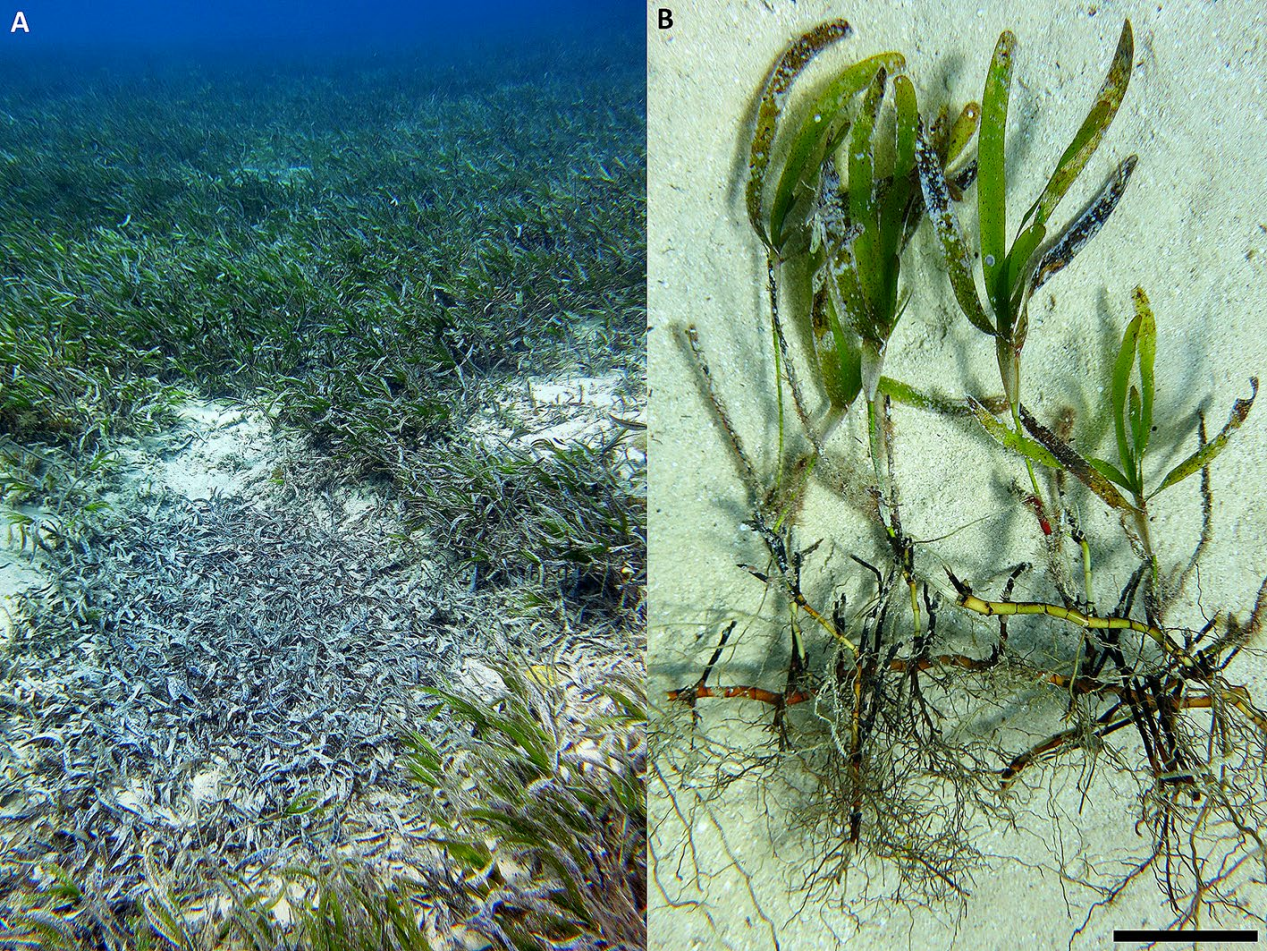
The investigated *Thalassodendron ciliatum* meadow (**A**) showing a patch of seagrass necromass in the foreground (not sampled). Next to nothing is known about the role of marine fungi in nutrient cycling in seagrass meadows. (**B**) Morphology of *T. ciliatum*, note its dense root system. Also note the coralligenous white sand in the background that forms the seabed at the investigated locality. Bar represents 5 cm

*Thalassodendron ciliatum* (Forsk.) den Hartog was sampled on February 25, 2019, at a site on the eastern coast of the Sinai Peninsula, between Nuweiba and Taba, close to Ras Shitan (= R. El Shetan, R. Shaitan, R. Shaitani, R. Shattein, etc.), Egypt (N29.1234, E34.6855; Fig. 1), where it grows in coralligenous white sand enriched with (broken) shells of various marine organisms (Foraminifera, Mollusca, etc.). In a dense monospecific meadow (Fig. 2A), intact samples (roots + rhizomes + shoots + leaves; Fig. 2B) of healthy-looking specimens were collected using scuba diving in ca. 6 m depth at three points ca. 3 m apart. The sampling depth was measured with a Freedom dive computer (Divesoft, Czechia), the underwater photos were taken with a Canon S100 camera in an underwater housing (Ikelite, USA). Upon delivery to the surface, the roots were separated, pooled, stored in 50% ethanol in seawater and transported to the laboratory where they were kept in a fridge at ca. 6 °C until used. A representative specimen (roots in 50% ethanol) was deposited in the Herbarium of the Institute of Botany, Czech Academy of Sciences, Průhonice, Czechia (PRA) under the accession number PRA-21596.

### Microscopy

All roots were initially screened with an Olympus SZX12 stereomicroscope and divided into two categories, namely 1/ without any visible fungal colonization and 2/ with visible fungal colonization on the root surface. To find out whether the root hairs’ presence is correlated with fungal colonization, fifty random root segments from both categories, each ca. 1 cm in length, were scored for the absence/presence of the root hairs. To find out whether the fungal colonization preferentially occurred in thinner (= terminal, younger) roots, the diameter of each root segment was measured at ten different points using QuickPHOTO MICRO ver. 3.2 (Promicra, Czechia), the average value was calculated in MS Excel, and the two categories were compared using the Kolmogorov-Smirnov two-sample test in STATISTICA 64 (Dell, USA). To screen possible intraradical fungal colonization, handmade longitudinal and transversal semithin sections of the roots from both categories were examined at high magnification (400×, 600×, and 1000×) with an Olympus BX60 microscope equipped with differential interference contrast. Microphotographs were taken with an Olympus DP70 camera and QuickPHOTO MICRO. In parallel, they were examined with a FEI Quanta 200 ESEM scanning electron microscope in the environmental mode at 275 Pa and − 12.5 to − 10 °C. Photo-documentation was adjusted for clarity and contrast as needed and assembled into figures using Paint.net ver. 4.3.12 (dotPDN LLC, Rick Brewster, and contributors).

### DNA isolation, amplification, cloning, and sequencing

**Fig. 3:**
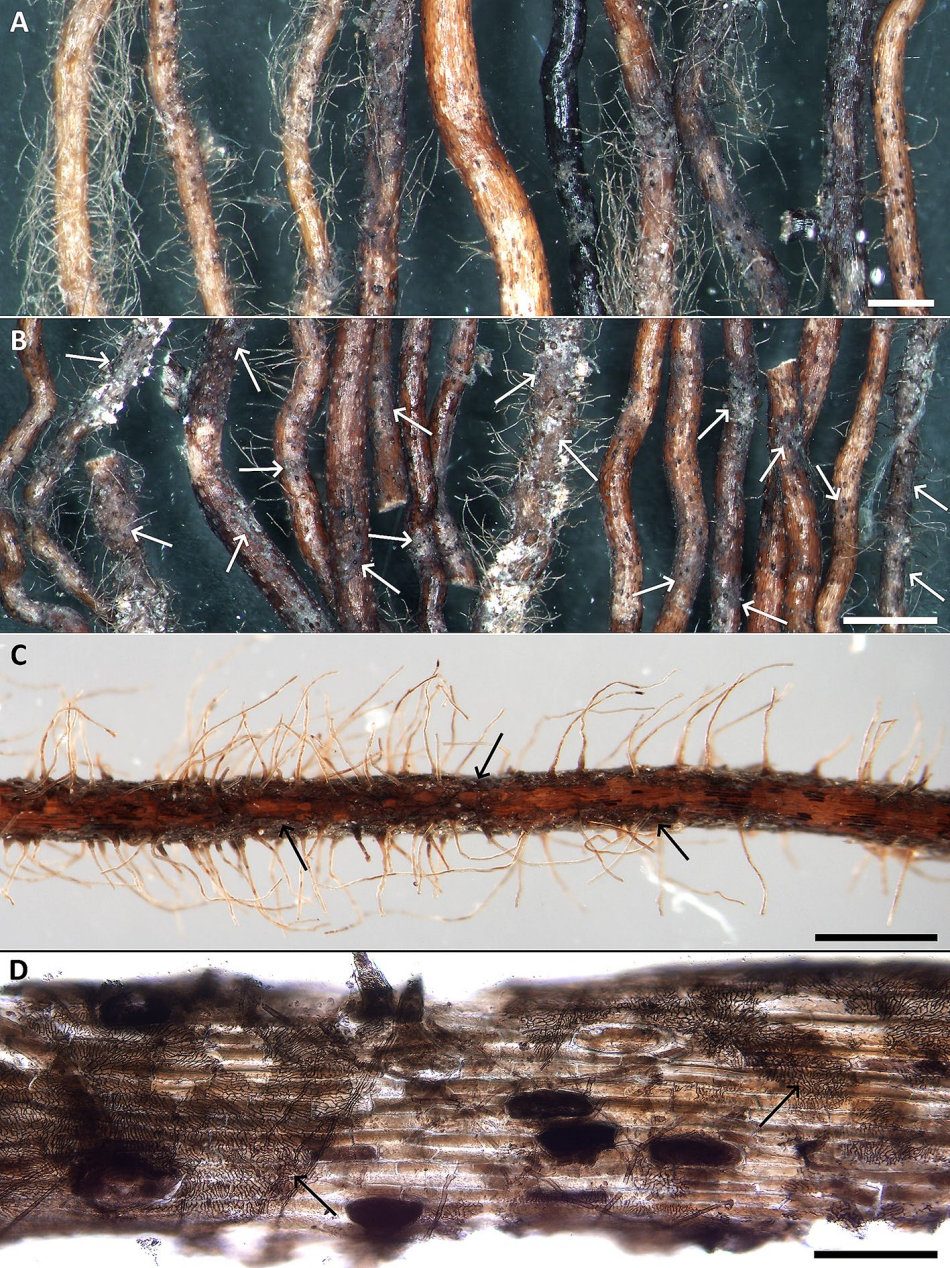
Morphology of *Thalassodendron ciliatum* roots and their superficial fungal colonization. (**A**) A random sample of roots differing in color, diameter, and presence/absence of the root hairs. Stereomicroscopy (SM), bar = 200 μm. (**B**) Selected roots displaying the characteristic fungal colonization on the root surface (arrows). SM, bar = 1000 μm. (**C**) A magnified view of a root colonized by dark mycelium (arrows), note the numerous root hairs. SM, bar = 500 μm. (**D**) A detail of the characteristic fungal colonization on the root surface (arrows). Light microscopy with differential interference contrast, bar = 100 μm

150 mg (fresh weight) of roots with visible superficial fungal colonization (Fig. 3A, B) were sonicated 1 × 5 min in 50% ethanol and 10 × 5 min in sterile Water for Molecular Biology (BioConcept, Switzerland) using an EMMI-05P ultrasonic bath (EMAG Technologies, Germany). Total DNA was extracted from the treated roots using a DNeasy Plant Mini Kit (Qiagen, Germany) according to the manufacturer’s instructions. The ITS rDNA region was amplified using the ITS1F + ITS4 primer pair (Gardes and Bruns 1993; White et al. 1990). The PCRs were performed in 25 µl reactions and contained 1x TopBio Plain PP Master Mix (TopBio, Czechia), each primer at 0.2 mM, 20 µg BSA, 1 mM MgCl2, and 25 ng of the isolated DNA. The cycling conditions were as follows: 5 min at 95 °C followed by 35 cycles of 95 °C for 30 s, 55 °C for 30 s and 72 °C for 1 min, and a final extension at 72 °C for 10 min.

Prior to cloning, the PCR products were excised from 1% agarose gels and purified with a Zymoclean Gel DNA Recovery kit (Zymoresearch, USA). The gel-purified fragments were cloned using the TOPO TA cloning kit (Invitrogen, USA) following the manufacturer’s instructions, but downscaled to half reactions. The colonies were transferred into 20 µl ddH20 and denatured at 95 °C for 10 min. They served as templates for subsequent PCR amplifications by the M13 forward and reverse primers (Invitrogen).

85 clones were sequenced at SEQme (Czechia) using the M13R primer (CAGGAAACAGCTATGACC). The obtained sequences were screened in Finch TV v. 1.4.0 (Geospiza, USA) and high-quality sequences were manually edited in the same software.

### Identification of the clones and trophic modes of the detected fungi

The edited sequences were aligned in Bioedit v. 7.1.8 (Hall 1999) and clustered at 99% similarity into operational taxonomic units (OTU) in TOPALi (Biomathematiscs & Statistics Scotland). When more than one, sequences within an OTU were aligned and a representative sequence was chosen based on quality and length. Representative sequences of each OTU were subjected to BLASTn searches (Zhang et al. 2000) in GenBank at NCBI (Sayers et al. 2019) as described in Vohník (2020). Their phylogenetic background was checked in Blast Tree View (https://www.ncbi.nlm.nih.gov/blast/treeview/treeview.cgi). Each OTU was assigned a species hypothesis (SH) in UNITE (Nilsson et al. 2019) and its tentative trophic mode was searched in The Faces of Fungi (Jayasiri et al. 2015), FUNGuild (Nguyen et al. 2016), and BioLib.cz (https://www.biolib.cz/cz/main/) databases. Representative sequences of each OTU were deposited in GenBank under the accession numbers OR392720-53. Fungal taxonomy followed MycoBank (https://www.mycobank.org).

## Results

### Microscopy

**Fig. 4:**
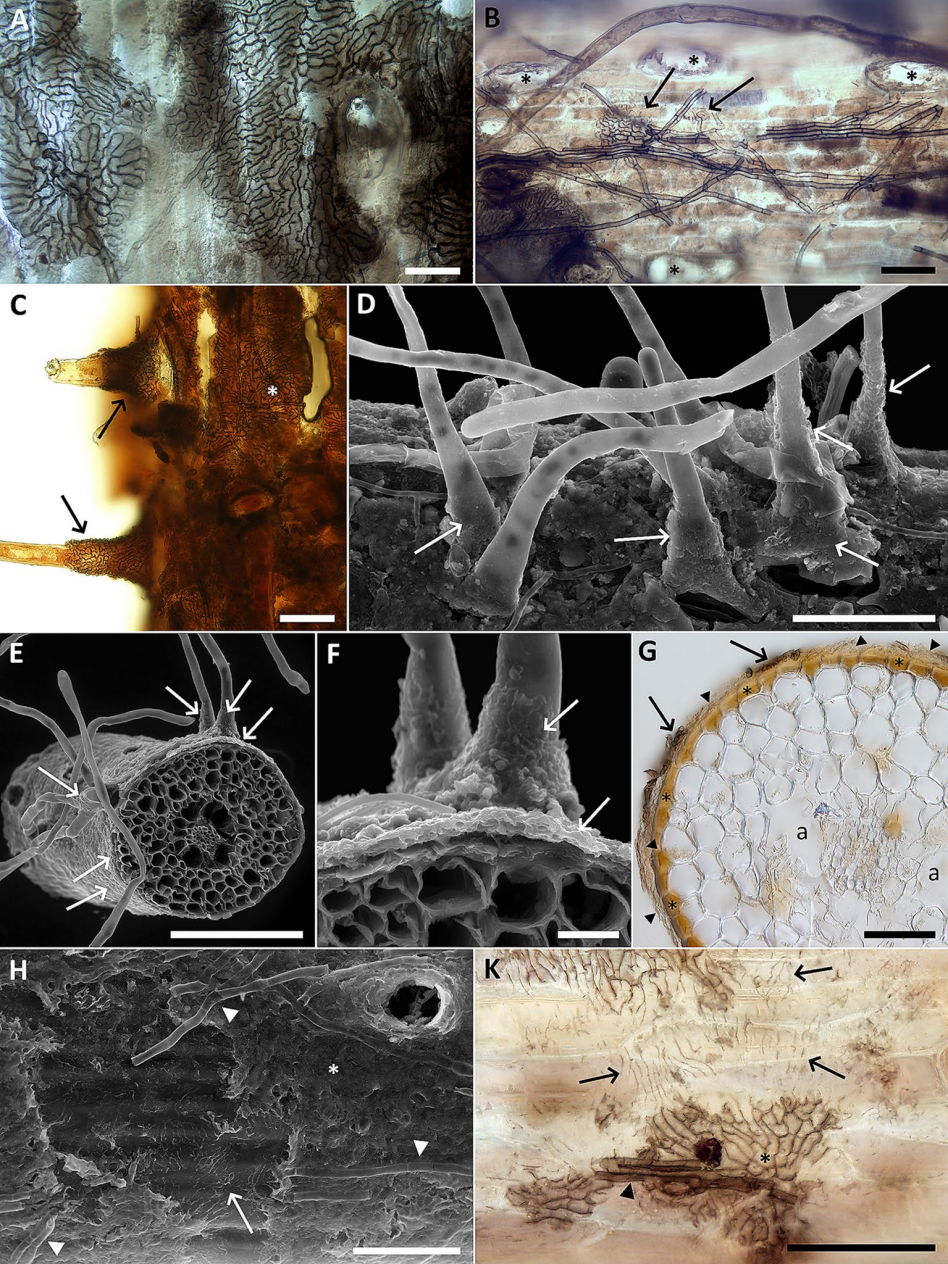
Typical features of the novel fungal symbiosis in the roots of *Thalassodendron ciliatum* (**A**) A discontinuous hyphal mantle (pseudoparenchymatous net) on the root surface. The cavity in the right side of the photo is due to a detached root hair. Light microscopy (LM) with differential interference contrast (DIC), bar = 20 μm. (**B**) Dark septate hyphae growing on the root surface either individually or parallelly attached to each other (resembling prosenchyma), eventually giving rise to the pseudoparenchymatous tissue (arrows). The cavities (asterisks) are after detached root hairs, also note a root hair in the upper part of the photo. LM with DIC, bar = 50 μm. (**C**) A hyphal mantle (asterisk) extending to the basal parts of the root hairs (arrows). LM with DIC, bar = 50 μm. (**D**) As in C. Scanning electron microscopy (SEM), bar = 100 μm. (**E**) A transversal section through a root with the root hairs and the characteristic fungal colonization on the root surface (arrows). Note no apparent intraradical fungal colonization. SEM, bar = 200 μm. (**F**) A detail from E. SEM, bar = 20 μm. (**G**) A transversal section through a root with rudiments of the hyphal mantles (arrows) accompanied by an unidentified substance, possibly of fungal origin (arrowheads). Note air lacunae (a) and the rhizodermal cells probably filled with phenolic compounds (some indicated by asterisks). LM with DIC, bar = 50 μm. (**H**) The hyphal mantle (asterisk) covering the root surface is detached in the left part of the photo, leaving imprints in the unidentified substance (arrow). Note some hyphae with visible septa on the mantle’s surface (arrowheads) and the cavity left after a detached root hair surrounded by fungal hyphae in the upper right corner of the photo. SEM, bar = 50 μm. (**K**) As in H. LM with DIC, bar = 50 μm

The root color varied from yellow/ochre (the youngest roots) to dark brown/black (the oldest roots) (Figs. 2B and 3A). The youngest roots typically possessed vigorous root hairs and these gradually disappeared with the root’s age (Fig. 3A). The root surface of darker/older roots was often densely colonized by DS fungal hyphae that formed discontinuous hyphal mantles resembling the pseudoparenchymatous nets formed by the terrestrial DSE or “mycélium en palmettes” (Figs. 3B–D and 4A and B) (Ducomet 1907; Renard et al. 2021). These typically originated from individual hyphae growing more or less linearly on the roots surface but later starting to produce shorter isodiametric cells that radially spread around (Fig. 4A, B). The mantles often covered the basal parts of the root hairs (Fig. 4C–F).

Transversal sections through the roots revealed no intraradical hyphal colonization (Fig. 4E–G). The mantles typically consisted of a single hyphal layer and were often accompanied by an unidentified substance (possibly of fungal origin) occurring between their abaxial surface and the root’s surface (Fig. 4G). When the mantle detached from the root, it left an imprint in the substance (Fig. 4H&K). The rhizodermal cells below the mantles were filled with a brownish substance (Fig. 4G), resembling the tannin cells formed in many ectomycorrhizae (Agerer 1987).

**Fig. 5:**
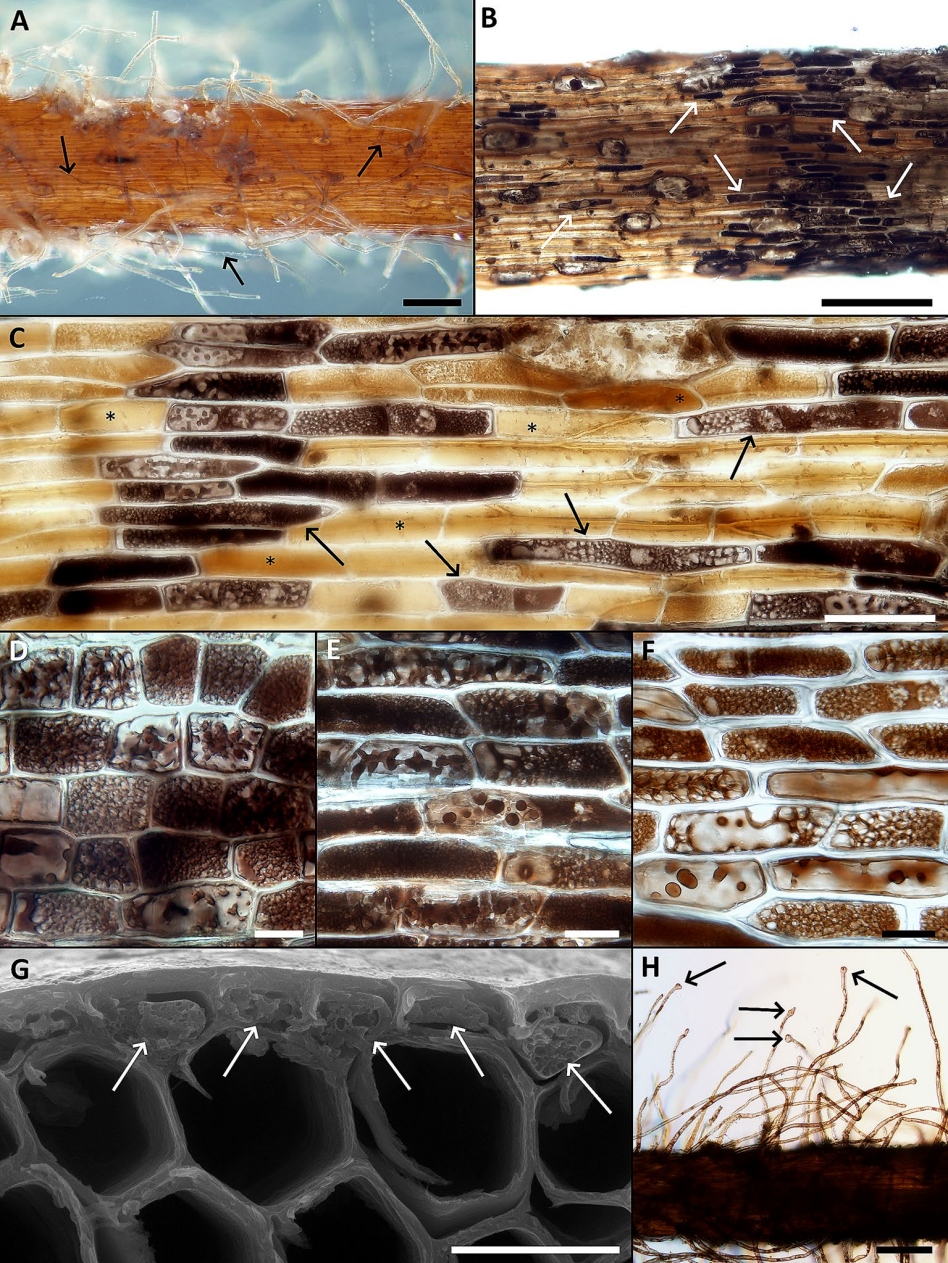
Some features of *Thalassodendron ciliatum* roots free of the novel fungal symbiosis. (**A**) Fungal hyphae (arrows) occurred on the root surface irrespective of the root age, absence/presence of the root hairs, and absence of the novel fungal symbiosis. Stereomicroscopy (SM), bar = 200 μm. (**B**) Older roots typically had a proportion of their rhizodermal cells filled with light- to dark-brown structures of varied shapes and these cells are interpreted here as the tannin cells (arrows). Light microscopy with differential interference contrast (DIC), bar = 200 μm. (**C**) Upon closer look, most rhizodermal cells were filled with a brownish substance (probably polyphenolic compound(-s), see Fig. 4G), resembling the tannin cells formed in many ectomycorrhizae (asterisks). It seemed like a transformation of this substance(-s) gives rise to the light- to dark-brown structures (arrows) also depicted in Fig. 5B. DIC, bar = 100 μm. (**D**, **E**, **F**) Details of the light- to dark-brown structures. DIC, bars = 20 μm. (**G**) The tannin cells (arrows). Scanning electron microscopy (SEM), bar = 20 μm. (**H)** The tips of some root hairs had a globular shape (arrows), remotely resembling the terminal swellings previously reported in the adhesive root hairs of the dominant Mediterranean seagrass *Posidonia oceanica*. SM, bar = 200 μm

Individual surface DS hyphae not producing mantles could be seen irrespectively of the roots age and the presence/absence of the root hairs (Fig. 5A). Some older roots, typically without visible DS fungal colonization and already without root hairs, had some of their rhizodermal cells filled with light- to dark brown structures of varied shapes (Fig. 5B–G) and these are interpreted here as polyphenolic substances occurring in the vacuoles of the tannin cells, similar to those occurring in the Mediterranean endemic seagrass *P. oceanica* (Lefebvre et al. 2023).

The root hairs’ presence was not correlated with visible DS fungal colonization as all screened roots in both categories (colonized vs. non-colonized) possessed root hairs, either intact or broken at their bases. The colonized roots (349.5 ± 54.2 μm, mean ± SD) had significantly smaller diameter (*p* < 0.001) than the non-colonized roots (505 ± 266.5 μm).

The tips of some root hairs had a globular shape (Fig. 5H) and in rare cases, they remotely resembled undeveloped terminal swellings previously reported in the adhesive root hairs of *P. oceanica* (Badalamenti et al. 2015; Kolátková and Vohník 2019). Alternatively, they might represent developing galls of an unidentified phytomyxid (Elliott et al. 2019; Kolátková et al. 2023).

### Identification of the clones and trophic modes of the detected fungi

The sequencing yielded 80 high-quality sequences and after editing, they were clustered into 34 OTU (Table 1). These belonged to Ascomycota (20 OTU/39 sequences), Basidiomycota (13/40), and Spermatophyta (1/1). In Ascomycota, Helotiales comprised 5 OTU/8 sequences, followed by Pleosporales (4/8), Hypocreales (3/12), Cladosporiales (3/5), Dothideales (2/3), Eurotiales (1/1), and Serinales (1/1). In Basidiomycota, Malasseziales comprised 5 OTU/26 sequences, followed by Polyporales (3/4), Tremellales (1/2), Russulales (1/2), and Agaricales (1/2). One ascomycetous and two basidiomycetous OTU could not be identified below the phylum level. The Spermatophyta OTU represented *T. ciliatum* (Table 1).

**Table 1:** Fungal and plant operational taxonomic units detected in this study

OTU (total seqs)	GenBank acc. #	Identity^1^	Taxonomy^2^	SH in UNITE^3^	FUNGuild trophic mode^4^	FUNGuild confidence ranking^4^
**1** (12)	OR392720	*Malassezia restricta*	Basidiomycota/Malasseziales	SH1102553.09FU *Malassezia restricta*	pathotroph	probable
**2** (9)	OR392721	*Malassezia* sp.	Basidiomycota/Malasseziales	-	pathotroph-saprotroph (genus)	probable
**3** (7)	OR392722	*Fusarium poae*	Ascomycota/Hypocreales	-	pathotroph	probable
**4** (3)	OR392723	*Trichoderma* sp.	Ascomycota/Hypocreales	SH1066571.09FU *Trichoderma erinaceum*	pathotroph-saprotroph-symbiotroph (genus)	probable
**5** (3)	OR392724	Helotiales sp.	Ascomycota/Helotiales	-	-	-
**6** (3)	OR392725	Pleosporales sp.	Ascomycota/Pleosporales	-	-	-
**7** (3)	OR392726	Pleosporales sp.	Ascomycota/Pleosporales	-	-	-
**8** (3)	OR392727	Tremellomycetes sp.	Basidiomycota	-	-	-
**9** (3)	OR392728	*Malassezia* sp.	Basidiomycota/Malasseziales	SH1102553.09FU *Malassezia restricta*	pathotroph-saprotroph (genus)	probable
**10** (2)	OR392729	*Aureobasidium pullulans*	Ascomycota/Dothideales	SH1240491.09FU *Aureobasidium pullulans*	pathotroph-symbiotroph	possible
**11** (2)	OR392730	*Cladosporium* sp.	Ascomycota/Cladosporiales	SH1309305.09FU *Cladosporium herbarum*	-	-
**12** (2)	OR392731	*Dioszegia crocea*	Basidiomycota/Tremellales	-	-	-
**13** (2)	OR392732	*Daedaleopsis confragosa*	Basidiomycota/Polyporales	SH1248911.09FU *Daedaleopsis confragosa*	pathotroph	probable
**14** (2)	OR392733	*Heterobasidion* sp.	Basidiomycota/Russulales	SH1236118.09FU *Heterobasidion annosum*	saprotroph (genus)	highly probable
**15** (2)	OR392734	*Strobilurus* sp.	Basidiomycota/Agaricales	SH1134327.09FU *Strobilurus esculentus*	saprotroph (genus)	probable
**16** (2)	OR392735	*Trichoderma* sp.	Ascomycota/Hypocreales	SH1066571.09FU *Trichoderma erinaceum*	pathotroph-saprotroph-symbiotroph (genus)	probable
**17** (2)	OR392736	*Cladosporium* sp.	Ascomycota/Cladosporiales	SH1309305.09FU *Cladosporium herbarum*	-	
**18** (2)	OR392737	*Crocicreas gramineum*	Ascomycota/Helotiales	-	saprotroph (genus)	probable
**19** (1)	OR392738	*Penicillium* sp.	Ascomycota/Eurotiales	SH0884485.09FU *Penicillium*	saprotroph (genus)	highly probable
**20** (1)	OR392739	Helotiales sp.	Ascomycota/Helotiales	SH0977021.09FU Helotiales	-	-
**21** (1)	OR392740	*Tetracladium maxilliforme*	Ascomycota/Helotiales	-	saprotroph (genus)	probable
**22** (1)	OR392741	*Tetracladium* sp.	Ascomycota/Helotiales	-	saprotroph (genus)	probable
**23** (1)	OR392742	Ascomycota sp.	Ascomycota	-	-	-
**24** (1)	OR392743	*Cladosporium* sp.	Ascomycota/Cladosporiales	SH1309305.09FU *Cladosporium*	-	-
**25** (1)	OR392744	*Stagonospora* sp.	Ascomycota/Pleosporales	-	pathotroph (genus)	probable
**26** (1)	OR392745	*Pyrenochaetopsis* sp.	Ascomycota/Pleosporales	-	pathotroph-saprotroph-symbiotroph (genus)	possible
**27** (1)	OR392746	*Malassezia* sp.	Basidiomycota/Malasseziales	-	pathotroph-saprotroph (genus)	probable
**28** (1)	OR392747	*Trametes versicolor*	Basidiomycota/Polyporales	SH1122493.09FU *Trametes versicolor*	saprotroph (genus)	highly probable
**29** (1)	OR392748	*Debaryomyces* sp.	Ascomycota/Serinales	SH1029444.09FU *Debaryomyces*	saprotroph (genus)	highly probable
**30** (1)	OR392749	*Lentinus brumalis*	Basidiomycota/Polyporales	SH1248739.09FU *Lentinus brumalis*	saprotroph (genus)	probable
**31** (1)	OR392750	Basidiomycetes sp.	Basidiomycota	-	-	-
**32** (1)	OR392751	*Thalassodendron ciliatum*	Alismatales/Cymodoceaceae	-	(photoautotroph)	-
**33** (1)	OR392752	*Malassezia* sp.	Basidiomycota/Malasseziales	SH1102553.09FU *Malassezia restricta*	pathotroph-saprotroph (genus)	probable
**34** (1)	OR392753	*Aureobasidium pullulans*	Ascomycota/Dothideales	SH1240491.09FU *Aureobasidium pullulans*	pathotroph-symbiotroph	possible

OTU = Operational Taxonomic Unit, SH = Species Hypothesis in the UNITE database for molecular identification of fungi (https://unite.ut.ee/)

^1^Based on BLAST searches in GenBank at NCBI (https://www.ncbi.nlm.nih.gov/genbank/) and BLAST Tree View as described in Materials and Methods

^2^Follows MycoBank (https://www.mycobank.org/) except OTU 32 that follows BioLib (https://www.biolib.cz/cz/main/)

^3^Shown only when sequence similarity ≥ 97%

^4^Follows FUNGuild (http://www.funguild.org/) at species or genus level (based on availability)

Because of their low taxonomic resolution, a trophic mode could not be attributed to 11 fungal OTU (Table 1). Most of the remaining fungal OTU were either saprotrophs (incl. wood saprobes) or pathotrophs (animal, human, or plant pathogens). None of the detected fungi were related to known mycorrhizal or DSE fungi, including *Pos. atricolor*, the dominant DSE of *P. oceanica*.

## Discussion

Prior to this study, only one seagrass has been reported to form a specific root-fungus symbiosis resembling those commonly occurring on dry land, and our observations thus extend the distribution and host taxonomic range of these associations for the NE Red Sea and another species in another seagrass family, respectively. However, unlike *P. oceanica* that is endemic to the Mediterranean, *T. ciliatum* is distributed across the Indo-Pacific (Green and Short 2003), making its symbiosis a potentially widespread phenomenon. The same is true for the more speciose Cymodoceaceae vs. Posidoniaceae that occur in the Caribbean, NW Africa, the Mediterranean, and most of the Indo-Pacific vs. being limited to the Mediterranean and SW to SE Australia (Angiosperm Phylogeny Website 2023). On the other hand, it is not known whether other members of Cymodoceaceae and Posidoniaceae form similar root-fungus symbioses. For example, despite that *Cymodocea nodosa* often co-occurs with *P. oceanica* and belongs to the same family as *T. ciliatum*, it does not seem to form any specific root-fungus symbiosis (Vohník et al. 2015).

### Root-fungus symbioses in *T. ciliatum* and *P. oceanica*

In addition to the differences in their distribution and taxonomy as well as anatomy and morphology of their roots, *T. ciliatum* and *P. oceanica* to some extent differ in anatomy and morphology of their root-fungus symbioses (Table 2). The most surprising difference is the absence of any visible intraradical hyphae in *T. ciliatum*, because in *P. oceanica* fungal hyphae often vigorously develop within the hypodermis (Vohník et al. 2015, 2019), forming the intracellular microsclerotia characteristic of DSE (e.g., Lukešová et al. 2015; Yu et al. 2001). In addition, while in *P. oceanica* fungal hyphae infrequently colonize the rhizodermal cells, these are fungus-free and filled with what appears as polyphenolic substances in *T. ciliatum* (cf. Cariello et al. 1979; McMillan 1984). Lastly, in *T. ciliatum* the DS fungal mantles cover the basal parts of the root hairs, a trait to our knowledge unknown in terrestrial roots, while these are typically absent in *P. oceanica* roots colonized by *Pos. atricolor* (Borovec and Vohník 2018). On the other hand, the mycobionts of both seagrasses form extensive hyphal mantles on the root surface (Vohník 2022; Vohník et al. 2017; this study) that are morphologically identical to those formed by DSE and certain ectomycorrhizal (EcM) fungi on the roots of compatible terrestrial plants (e.g., Kaldorf et al. 2004). Intriguingly, similar structures called *mycélium en palmettes* (Ducomet 1907) are formed by some foliicolous Dothideomycetes on the leaf and twig cuticle where the respective mycobionts eventually form thyriothecia (Renard et al. 2021). However, these have not been detected on the roots investigated here.

**Table 2:** Comparison of the seagrasses *Thalassodendron ciliatum* and *Posidonia oceanica* with focus on their interactions with fungi

Seagrass species (family in Alismatales)	Distribution	Hypodermis	Root hairs	Tannin cells in the roots	Main fungal partner	Other fungal partners	Surface hyphal mantles	Intraradical colonization	Fungal interaction with root hairs
*Thalassodendron ciliatum* (Cymodoceaceae)	Indo-Pacific	no	yes, often abundant in adults	yes (in rhizodermis)	unknown	see Table 1	yes	no	dense hyphal mantles covering the root hairs’ bases
*Posidonia oceanica* (Posidoniaceae)	Mediterranean Sea (endemic, remaining *Posidonia* species in southern Australia)	yes	abundant in seedlings, mostly absent in adults	no	*Posidoniomyces atricolor* (Aigialaceae, Pleosporales)	lulworthioid fungi (Lulworthiales), other marine fungi (see Introduction for references)	yes	yes (intracellular microsclerotia in hypodermis, intracellular hyphae in rhizodermis, intercellular hyphae in rhizodermis and hypodermis)	negative correlation with the root hairs’ presence

### Fungal partners in *T. ciliatum* and *P. oceanica*

It has been repeatedly shown that *Pos. atricolor* mycelium develops from the intracellular microsclerotia occurring in the hypodermis of *P. oceanica* (Vohník et al. 2016, 2019; Vohník 2021, 2022) and *Pos. atricolor* has been detected in the terminal roots of *P. oceanica* adults at every sampled locality in the whole N Mediterranean (M. Vohník, unpublished data). At the same time, *Pos. atricolor* has not been detected in any other host or substrate nor by any other research team. In addition, the mycobiota of *P. oceanica* roots typically comprises lulworthioid fungi (Lulworthiales) (Torta et al. 2022; Poli et al. 2021; Vohník et al. 2016, 2017) but their functioning is unclear (Vohník 2022). To our surprise, none of these fungi nor their relatives were detected in the investigated *T. ciliatum* roots. This might be due to their genuine absence, the different detection methods used in this (cloning) and the previous (culturing and high-throughput sequencing) studies, or incompatibility with the primers used in this study (cf. (Vohník et al. 2012).

To our knowledge, this is the first report on the mycobiota associated with the roots of *T. ciliatum*. In general, the most surprising results were the relatively high incidence of basidiomycetes and the dominance of saprotrophs and pathotrophs, both to a large extent due to the high incidence of *Malassezia* spp. (Table 1). *Malassezia* are ecologically versatile yeasts known from both terrestrial and marine environments and they occur on such diverse substrates as corals, deep-sea vents, and mammal skin (e.g., Amend 2014). They are commensals, pathogens, and saprobes and only rarely form hyphae (e.g., Saadatzadeh et al. 2001). It is thus not probable that they form the DS hyphal mantles characteristic of the novel root-fungus symbiosis reported here. Similarly, none of the six non-*Malassezia* OTU with ≥ 3 sequences seem like probable candidates for the observed colonization pattern. For example, *Fusarium poae* is a known plant pathogen (e.g., Stenglein 2009), *Trichoderma* are mycoparasites, saprobes, and pathogens (e.g., Williams et al. 2003) and none of them typically produce melanized hyphae (Podgórska-Kryszczuk et al. 2022; Wang et al. 2016).

Four OTU belonged to Pleosporales but none to Aigialaceae, i.e., the same family as *Pos. atricolor*. OTU-6/Pleosporales sp. grouped with *Stagonospora* sp. (GenBank OM337558, Massarinaceae), *Phaeosphaeriopsis* sp. (HQ630983, Phaeosphaeriaceae) obtained from *Miscanthus giganteus* (Poales: Poaceae) from Illinois, USA (Shrestha et al. 2011), and *Didymocyrtis cladoniicola* (LT796877, Phaeosphaeriaceae) from USA, all with > 99% sequence similarity. *Stagonospora* are probable plant pathogens (e.g., Solomon et al. 2006), Phaeosphaeriaceae are pathogenic, saprobic, or hyperparasitic mostly on monocotyledons and especially Poaceae (Hyde et al. 2013), and *D. cladoniicola* is a probable lichen parasite (Lawrey and Diederich 2018). While no GenBank entry displayed > 90% sequence similarity with OTU-7/Pleosporales sp., OTU 25 belongs to *Stagonospora* sp. and OTU 26 to *Pyrenochaetopsis* sp. (Pyrenochaetopsidaceae), displaying 99.4% sequence similarity with *Pyrenochaetopsis* sp. PG293 (AB916515) from a bird feather from Svalbard (Singh et al. 2016). *Pyrenochaetopsis* comprises commensals, plant endophytes and pathogens, and saprobes occurring in animals, humans, plants, soil, and water (e.g., Špetík et al. 2021).

When searching for the mycobiont forming the novel symbiosis one should not discriminate fungi related to known saprobes and/or pathogens. For example, *Pos. atricolor* represents the only biotrophic lineage within the otherwise saprobic Aigialaceae (Vohník et al. 2019; Suetrong et al. 2009), certain mycorrhizal fungi also inhabit the soil and wood as saprobes (Rice and Currah 2006; Fehrer et al. 2019; Kolařík and Vohník 2018; Vohník and Réblová 2023), etc. Likewise, not all fungi belonging to genera, families, and orders comprising widespread plant endophytes necessarily share this trait, an excellent example being Helotiales (e.g., Zijlstra et al. 2005). In our study, five OTU belonged to Helotiales: OTU 5 and 18 displayed affinities to *Crocicreas gramineum* (Helotiaceae) which is a saprobe on plant debris and leaves, especially on Poaceae (Domínguez 2017). OTU 20 clustered with several *Lemonniera* sp. (Discinellaceae) that are saprotrophs on dead plant material (Ekanayaka et al. 2019). Finally, OTU 21 and 22 belonged to *Tetracladium* (Helotiales inc. sed.) which comprises aquatic hyphomycetes sometimes colonizing plant roots as endophytes (Selosse et al. 2008). Under these circumstances, we cannot be sure if we detected the mycobiont forming the novel symbiosis nor what is its taxonomy. Nevertheless, despite the limited sampling our study reveals a relatively high fungal diversity associated with the roots of a common Indo-Pacific seagrass that begs further investigation, a situation similar to many freshwater plants (e.g., Kohout et al. 2012).

### Functioning of DS fungal associations in seagrasses

There is an ongoing debate about the role of DSE in plant ecology and physiology and it seems that they can be beneficial, neutral, or detrimental associates of terrestrial plants, depending on the phytobiont and mycobiont taxonomy and ontogeny as well as a wide array of environmental conditions (Newsham 2011; Reininger and Sieber 2012; Usuki and Narisawa 2007; Vohník et al. 2003; Mayerhofer et al. 2013). On the other hand, virtually nothing is known about the functioning of DSE/DS mycobionts in seagrasses and changing this will require manipulative monoxenic inoculation experiments, isotopic studies, and genome analyses. In *P. oceanica*, there is an ontogenetic shift from the seedlings whose roots possess dense root hairs but lack the DSE symbiosis to the adults mostly without root hairs but regularly forming the DSE symbiosis, which is similar to non-mycorrhizal vs. EcM roots (Borovec and Vohník 2018). However, it is unknown whether this shift is directly related to *Pos. atricolor* and in *T. ciliatum*, the mycobiont’s presence does not seem to be in any relationship with the presence of the root hairs.

Although indirect, this study provides two important hints on the functioning of the novel symbiosis in *T. ciliatum*. First, the observation that the hyphal mantles stay on the root surface without visible intraradical colonization suggests that the mycobiont lives as a fungal epiphyte. Epiphytism in fungi is an ancient widespread trait that has evolved independently in several ascomycetous lineages (Hongsanan et al. 2016) but typically concerns plant aboveground organs, especially the leaves, and to our knowledge has never been reported from the roots. While it is unclear whether any parallels can be drawn between terrestrial leaf and marine root fungal epiphytes, they might protect the roots from bacterial, fungal, and viral pathogens, damage caused by herbivores, osmotic stress, etc. In this context, it is interesting to note that older roots typically without fungal colonization had their rhizodermal cells filled with light- to dark brown structures of varied shapes, possibly formed by polyphenolic substances that protect the roots from the stresses listed above (Kumar et al. 2020). Since these were less intense in the colonized roots, one might hypothesize that the hyphal mantles take over their protection role, eventually saving the seagrass the energy and metabolites necessary to produce these substances. Second, the DS fungal colonization was more frequent in thinner terminal roots that are typically the sites of nutrient uptake, indicating a possible role of the mycobiont in the seagrass nutrition, as already hypothesized for *Pos. atricolor* in the dominant Mediterranean seagrass *P. oceanica* (Vohník et al. 2015). However, the apparent epiphytic nature of the novel symbiosis hints against a direct nutrient transfer between the mycobiont and its host seagrass. On the other hand, some fungi may benefit their plant partners without forming intraradical mycorrhizal structures, as experimentally demonstrated by Kariman et al. (2014). In any case, further research is needed to test these hypotheses.

## Conclusions

Our results indicate that specific root-fungus symbioses in seagrasses might be more frequent than previously thought, being so far confirmed in two highly productive seagrasses from two different families inhabiting two different regions. While their functioning and significance are currently unknown, they appear in healthy-looking terminal roots (i.e., the sites of the nutrient uptake from the seabed) of healthy-looking hosts. The two so far known symbioses are formed by mycobionts with relatively thick melanized hyphae that produce mantles on the root surface that might confer protection against herbivores and pathogens. Melanin slows down decomposition of the fungal mycelium and hence also the colonized terrestrial roots (Langley et al. 2006). If similar is true for some seagrasses (e.g., *P. oceanica* and *T. ciliatum*), their root-symbiotic fungi would significantly contribute to the accumulation and stabilization of blue carbon buried in the seabed below the respective seagrass meadows.

## Data Availability

Sequences generated in this study are available in GenBank at NCBI under the accession numbers OR392720-53. A *Thalassodendron ciliatum* root specimen is deposited in the Herbarium of the Institute of Botany, Czech Academy of Sciences, Průhonice, Czechia (PRA) under the accession number PRA-21596.
